# Home-learning environment and cognitive and academic outcomes among children aged 4–8 years: A cross-sectional study from South India

**DOI:** 10.1016/j.dialog.2025.100238

**Published:** 2025-09-06

**Authors:** Eunice Lobo, Debarati Mukherjee, Pradeep Kumar Choudhury, Giridhara Rathnaiah Babu, Prashanth Nuggehalli Srinivas, Onno C.P. van Schayck

**Affiliations:** a Indian Institute of Public Health - Bengaluru, Public Health Foundation of India, Epidemic Diseases Hospital premises, Near Swami Vivekananda Metro Station, Old Madras Road, Bengaluru 560038, Karnataka, India; b PHFI Centre for Developmental and Lifecourse Research, Epidemic Diseases Hospital premises, Near Swami Vivekananda Metro Station, Old Madras Road, Bengaluru, Karnataka 560038, India; c Centre for Health Systems, Institute of Public Health Bengaluru, 3009, II-A Main, 17th Cross, Krishna Rajendra Rd, Banashankari Stage II, Bengaluru, Karnataka 560070, India; d Department of Family Medicine, Care and Public Health Research Institute (CAPHRI), Maastricht University, P.O. Box 616, Maastricht, 6200, MD, the Netherlands; e Zakir Husain Centre for Educational Studies, School of Social Sciences, Jawaharlal Nehru University, New Delhi 110067, India; f Department of Population Medicine, College of Medicine, QU Health, Qatar University, P.O. Box: 2713, Doha, Qatar

**Keywords:** Family care indicators, Urban poor, COINCIDE study, Stimulation, Home-learning, Child development

## Abstract

**Purpose:**

Home-learning environment is critical for cognitive and academic outcomes; yet its impact during the 4–8 years' period remains underexplored, especially in the Global South. This study examines the relationship between the home-learning environment and children's fluid intelligence and early language numeracy outcomes in urban poor households in Bangalore, South India.

**Methods:**

We analysed data from 940 mother-child dyads from the MAASTHI birth cohort when children were 4–8-years old. The Family Care Indicators (FCI) tool assessed the home-learning environment, Raven's Coloured Progressive Matrices (RCPM) measured children's fluid-intelligence, and the preschool Annual Status of Education Report (ASER) tool measured literacy-numeracy skills. Multilevel linear regression models, adjusted for household, maternal, and child factors, were used to examine the associations.

**Results:**

Higher levels of parental education, maternal Intelligence Quotient, and lower maternal depressive symptoms were significantly associated with better cognitive and early language outcomes. A stimulating home-learning environment characterized by the availability of ≥6 age-appropriate books, higher levels of caregiver engagement, and higher overall home environment scores (FCI-Total) was linked to better non-verbal fluid intelligence and early language scores during 4–8 years of age. However, these factors were not significantly associated with numeracy.

**Conclusion:**

This study underscores the sustained benefits of a stimulating home-learning environment in urban poor settings on children's cognitive and academic outcomes between 4 and 8 years of age. Our results reinforce the need for interventions that promote caregiver engagement and access to a variety of books and toys to optimize child outcomes in marginalized settings.

## Introduction

The first decade of life is critical for equipping children with cognitive, language, social-emotional, and academic skills, which shape health and economic outcomes throughout life [1,2]. However, 250 million under-5 children in low- and middle-income countries (LMICs), constituting 43 % of the world's under-five population, are developing sub-optimally [2]. To address this, the World Health Organization (WHO), World Bank, UNICEF, and other global stakeholders launched the Nurturing Care Framework (NCF) for Early Childhood Development in 2018. This framework emphasizes five essential areas to optimize children's development: good health, adequate nutrition, safety and security, responsive caregiving, and opportunities for early learning [3]. Notably, the home environment is the primary setting determining the quality of these exposures for children.

Research, primarily from the Global North, underscores the crucial influence of cognitive stimulation and early learning opportunities in shaping children's cognitive and academic outcomes [4,5]. Activities such as playing with a variety of toys, reading or being read age-appropriate books, singing or listening to children's songs, and outdoor play fosters early language skills, expands vocabulary, and enhances memory and attention [6]. Reading with caregivers promotes parent-child bonding and socio-emotional development [7], while toys provide opportunities for creative and imaginary play, thereby supporting problem-solving skills and encouraging social interactions while playing in groups [8]. Therefore, providing children with diverse learning opportunities through books, toys, and caregiver engagement can profoundly impact holistic development in children and set the groundwork for lifelong learning, as emphasized in the fourth Sustainable Development Goal [9].

The quality of the home-learning environment is heavily influenced by household-level socio-economic status (SES) [10,11]. Caregivers from low-income households often struggle to provide a responsive and stimulating learning environment due to food insecurity, income instability, unstable homes, etc. [12]. Despite these challenges, an accumulating body of evidence shows that early childhood stimulation has lasting positive impacts on cognitive and social-emotional development in children from LMICs. For example, a recent study from Pakistan demonstrated that psychosocial stimulation at home is strongly associated with school readiness [13]. Similarly, home stimulation was associated with improved cognitive outcomes in Ugandan children living in extreme poverty [14].

**Fig. 1:**
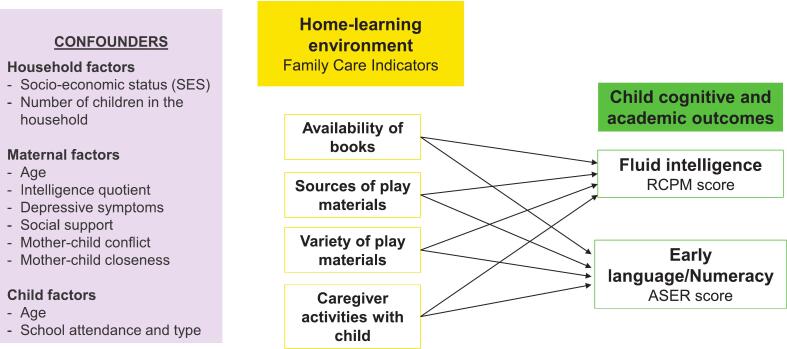
Conceptual framework for the role of the home-learning environment in shaping children's cognitive and academic outcomes during 4–8 years of age.

Children in urban poor settings in India face unique challenges, including limited access to open and safe spaces for play, reduced opportunities for peer interaction, and increased time spent at home, coupled with limited interaction with primary caregivers due to parental work pressures and low social and community support. However, a detailed characterization of the home-learning environment, and its relationship with cognitive and academic abilities beyond the first three years *especially from 4 to 8 years* is under-explored, making it challenging to identify context-specific risk and protective factors in India. This study aims to examine the association between the home-learning environment and cognitive and academic outcomes (fluid intelligence, early language, and numeracy) in 4–8-year-old children from urban poor households in Bangalore, South India. We hypothesize that the presence of (i) *more age-appropriate books; (ii) greater variety of play materials; (iii) more caregiver engagement will be associated with better child outcomes in urban poor households in South India (*Fig. 1*).*

## Methods

### Study design, participants, sample size

This is a cross-sectional study that utilized data from the COINCIDE study [15], that aims to assess the nutritional, psychosocial, and environmental determinants of children's neurodevelopment and mental health. The COINCIDE study is nested within the Maternal Antecedents of Adiposity and Studying the transgenerational role of Hyperglycaemia and Insulin (MAASTHI) birth cohort [16,17], a longitudinal study that recruited 2962 pregnant women from public hospitals in Bengaluru, South India, between 2016 and 2019 to understand the transgenerational role of increased glucose levels or hyperglycaemia and other nutrients and psychosocial environment, on the risk of childhood obesity, as an early marker of chronic diseases. In 2023–24, we followed-up 989 mother-child dyads from MAASTHI when children were 3–8-years-old. Since our outcome assessment tools were only applicable to children ≥4 years, we excluded *N* = 49 children under 4 years, yielding a final analytic sample of 940 mother-child dyads. The socio-demographic characteristics of these participants (*N* = 940) were similar to those of those who were not followed up (*N* = 2022, Supplementary Table 1). We used the Strengthening the Reporting of Observational Studies in Epidemiology (STROBE) guidelines to report our methods and findings (see Supplementary checklist 1).

Trained assessors collected data on the REDCap mobile application [18] between April 2023 and April 2024 in participants' households. Visits were typically scheduled during the afternoons after children returned from school. Inclusion criteria were: (a) no history of neurological, vision, hearing, or motor impairments in the child as reported by the primary caregiver; (b) willingness to provide written informed consent. Field data collectors scheduled appointments for the home visit in advance at a time convenient for both the mother and child through phone calls, and obtained written informed consent from the primary caregiver or guardian upon arrival. Assessments were conducted with two assessors working in pairs: one collected data from the primary caregiver, typically the mother, though in her absence, data was collected from grandparents or the father. The second assessor facilitated performance-based assessments to measure cognitive and academic outcomes in children. Approximately 10 % of assessments were supervised by Senior Research Associates who ensured protocol adherence and provided feedback if errors were noted. The average inter-rater reliability between assessors, calculated using Cohen's kappa statistic, was high (0.97).

### Measurements (Fig. 1)

#### Exposure assessment: Home-learning environment

The Home Observation for Measurement of the Environment (HOME) inventory is one of the most commonly used tools to measure the quality of the home environment and caregiving practices. However, due to its observational nature and considerable time investments, a shorter parent-report version – the Family Care Indicators (FCI) - has been adapted for use in large-scale epidemiological surveys across culturally diverse global settings [19]. We used the FCI tool to collect data on the availability of age-appropriate books, sources, and varieties of play materials available at home, and types of activities caregivers engaged with the index child in the past three days before the assessment (Fig. 1). The primary measure of the FCI tool is the total score, which reflects the overall home-learning environment, with higher scores indicative of a better quality of the home-learning environment. We also considered domain scores (number of books, sources, and varieties of toys, types of caregiver activities) in the analysis. Further details are provided in Supplementary data 1.

#### Outcome assessment: Cognitive and academic outcomes

Fluid intelligence (abstract reasoning and non-verbal problem-solving skills) was measured using the Raven's Coloured Progressive Matrices (RCPM), a validated intelligence quotient test for children aged 4–11 years with norms available for India. [20]

Early language and numeracy skills were assessed using the preschool version of the Annual Status of Education Report (ASER), a standardized and validated tool for 4–8-year-old children in India. [21] More details on specific items and scoring are presented in Supplementary data 1.

#### Confounding variables

Regression models were adjusted for several confounders identified from the literature, listed below and detailed in Supplementary data1.

*Child factors:* child age, school attendance, and school type (none, private, or public).

*Maternal factors:* maternal age, maternal IQ (Raven's Standard Progressive Matrices), depressive symptoms (Edinburgh Postnatal Depression Scale [EPDS] with a cut-off of ≥13 considered for having depressive symptoms [22], maternal social support (Maternal Social Support scale [MSS]). Mother-child relationship was assessed using the Child-Parent Relationship Scale (CPRS), categorized into four levels based on median splits: (i) low conflict-high closeness, (ii) low conflict-low closeness, (iii) high conflict-low closeness, and (iv) high conflict-high closeness.

*Household factors:* socioeconomic status (SES) and number of children in the household, excluding the index child.

### Statistical analysis plan

Data were analysed using the R statistical software version 4.2.3 (Core Team, Vienna, Austria). Descriptive statistics were used to report the socio-demographic characteristics of participants and characterize the exposure, outcome, and confounding variables. Continuous variables were summarized as mean and standard deviation or medians and interquartile ranges (IQR) if the distribution was skewed. Initial exploratory analysis revealed a strong relationship between children's age and early language-numeracy outcomes, and mean child age varied across different levels of parental factors (for example, more educated mothers had younger children). Hence, we used univariable models adjusted for child age to assess the relationship between each exposure and children's early language-numeracy outcomes to avoid confounding by child age. This was not required for the RCPM outcome, as the scaled score and percentile rank are already age-standardized using Indian norms.

To test our hypothesis that the higher number of age-appropriate books, greater variety of play materials, and higher caregiver engagement are associated with better child outcomes in urban poor households, we used multilevel linear regression models to examine these associations while accounting for data clustering within geographical zones and adjusting for potential confounders. As this is a cross-sectional study, results should be interpreted as associations, not evidence of causality. Relationships between specific domains and overall home-learning environment with child cognitive and academic outcomes were estimated using multilevel linear regression models with geographical clustering within urban Bangalore as the random effect. Models were adjusted using confounding variables delineated in the directed acyclic graph presented in Supplementary Fig. 1. A hierarchical approach was followed with models progressively adjusted for child age and household factors (Model 1), Model 1 + maternal factors (Model 2), and Model 2 + child school attendance and type (Model 3). Outcome measures were scaled (z-scores) to allow direct comparisons of the results across the three outcome measures (RCPM percentile rank, ASER early language score, ASER numeracy score). Results were reported for the primary exposure measure (FCI domain and total scores) treated as both continuous and categorical variables. Statistical significance was considered at *p* < 0.05.

### Ethical considerations

This study was conducted in accordance with the principles of the Declaration of Helsinki. Ethical approval was obtained from the Institutional Ethics Committee of the Indian Institute of Public Health-Bengaluru, Public Health Foundation of India (IIPHHB/TRCIEC/214/2021). Participation was voluntary, with the option to withdraw or refuse to respond to any item they were not comfortable answering. No monetary compensation was provided; instead, children were gifted an art kit as a token of appreciation.

## Results

### Study population characteristics (Table 1) (*N* = 940)

The median child age was 4.95 years (interquartile range [IQR] 1.65), with most children (*n* = 679; 72.2 %) being between four and six years old. Sex distribution was almost even, with 50.4 % being girls. Most children were either first-born (*n* = 419; 44.6 %) or second-born (*n* = 388; 41.3 %), and attended private schools (75.1 %; *n* = 706). 7.2 % were yet to enrol in any school, and 51.9 % played video games on smartphones or tablet devices more than three times a week.

**Table 1:** Characteristics of the study population in the MAASTHI cohort (*N* = 940).

*Variable*	N (%)
***Age (in years)*** 4–5.9 6–8 ***Median age in years (IQR)***	679 (72.2 %) 261 (27.8 %) 4.95 (1.65)
***Sex*** Boy Girl	466 (49.6 %) 474 (50.4 %)
***Birth order of the index child*** First born Second Third > 4 ***Missing***	419 (44.6 %) 388 (41.3 %) 99 (10.5 %) 31 (3.2 %) 3 (0.3 %)
***Type of school attendance*** Not yet enrolled Government school Private school ***Missing***	68 (7.2 %) 165 (17.6 %) 706 (75.1 %) 1 (0.1 %)
***Smartphone or tablet usage*** Nil Rare (About once a month) Moderate (About once a week) Often (> 3 times a week) ***Missing***	83 (8.8 %) 119 (12.7 %) 188 (20.0 %) 488 (51.9 %) 62 (6.6 %)
***Respondent relationship with child*** Mother Other caregiver ***Missing***	913 (97.1 %) 26 (2.7 %) 1 (0.1 %)
***Mother's age at assessment (in years)*** 20–30 31–40 41–50 ***Median age in years (IQR)*** ***Missing***	481 (51.2 %) 416 (44.3 %) 31 (3.3 %) 29.7 (6.83) 11 (1.1 %)
***Mother's education*** Illiterate/No schooling Standard 1–4 Standard 5–8 Standard 9–12 Diploma Graduate Post-graduate ***Missing***	26 (2.7 %) 10 (1.0 %) 206 (21.9 %) 565 (60.1 %) 33 (3.5 %) 86 (9.1 %) 10 (1.0 %) 4 (0.4 %)
***Mother IQ*** *Median RSPM score (IQR)* *Range* ***Missing***	30 (8) 12–47 108 (11.5 %)
***Father's education*** Illiterate/No schooling Standard 1–4 Standard 5–8 Standard 9–12 Diploma Graduate Post-graduate ***Missing***	72 (7.6 %) 38 (4.0 %) 231 (24.6 %) 465 (49.5 %) 39 (4.1 %) 72 (7.6 %) 11 (1.2 %) 12 (1.3 %)
***Mother's occupation*** Unemployed or Homemaker Unskilled worker Semiskilled worker Skilled worker Professional ***Missing***	648 (68.9 %) 128 (13.6 %) 50 (5.3 %) 32 (3.4 %) 74 (7.9 %) 4 (0.4 %)
***Father's occupation*** Unemployed Unskilled worker Semiskilled worker Skilled worker Professional ***Missing***	9 (0.95 %) 169 (18.0 %) 125 (13.3 %) 426 (45.3 %) 192 (20.4 %) 12 (1.3 %)
***Maternal depressive symptoms (EPDS scores)*** EPDS score ≤ 13 EPDS score > 13 ***Median EPDS score (IQR)*** *Range* ***Missing***	845 (89.9 %) 79 (8.4 %) 5 (8) 0–29 16 (1.7 %)
***Maternal social support*** ***Median MSS score (IQR)*** *Range* ***Missing***	23 (12) 0–36 8 (0.8 %)
***Mother-child relationship (CPRS scores)*** Median Conflict score (IQR) Range [Conflict score] Median Closeness score (IQR) Range [Closeness score] ***Missing*** Low-conflict & High closeness Low-conflict & Low closeness High-conflict & High closeness High-conflict & Low closeness	20.5 (8) 8–36 35 (4) 19–35 4 (0.4 %) 255 (27.1 %) 212 (22.6 %) 243 (25.9 %) 225 (23.9 %)
***Number of children in the household other than index child*** 0 1 2 ≥ 3 ***Missing***	84 (8.9 %) 384 (40.9 %) 335 (35.6 %) 134 (14.2 %) 3 (0.3 %)

IQR – Interquartile range; EPDS -Edinburgh Postnatal Depression Scale; MSS – Maternal Social Support; CPRS –Child Parent Relationship Scale.

Mothers were the primary respondents to the parent-reported questionnaires (97.1 %; *n* = 913). Their median age at the time of assessment was 29.7 (IQR 6.83) years, 60.1 % had completed high-school education (*n* = 565), and 68.9 % (*n* = 648) were homemakers. Median maternal IQ score measured using the Raven's Standard Progressive Matrices was 30 (IQR 8). Fathers' educational levels were comparable, with 49.5 % (*n* = 465) having completed high school. Most engaged in skilled work (45.3 %) and professional services (20.4 %).

Maternal mental health assessed using EPDS reported a median score of 5 (IQR 8), with 79 mothers (8.4 %) scoring 13 or above, indicative of depressive symptoms. Median maternal social support score was 23 (IQR 12). Median parent-child conflict score (possible score range: 8–40) was 20.5 (IQR 8), and closeness score (possible score range: 7–35) was 35 (IQR 4). Most households had one (*n* = 384; 40.9 %) or two (*n* = 335; 35.6 %) children other than the index child.

The age-adjusted RCPM percentile rank measuring fluid intelligence demonstrated large variation, ranging from 0.1 to 99.9 (possible score range: 0–100), with a median score of 37 (IQR 47). Early language scores ranged from 1 to 9 (possible score range: 0–9), with a median score of 5 (IQR 1), while numeracy scores (possible score range: 0–14) ranged from 0 to 14, with a median score of 5 (IQR 4).

RCPM percentile rank showed a weak correlation with early language (**ρ** = −0.13, *p* < 0.0001) and numeracy scores (ρ = −0.19, p < 0.0001). However, early language and numeracy scores showed moderate levels of correlation (**ρ** = 0.68, *p* ≤0.0001), highlighting that fluid intelligence and early language-numeracy outcomes are distinct skillsets. However, early language and numeracy skills are closely related.

### Univariable associations between child, caregiver, and household characteristics with child cognitive and academic outcomes

We conducted univariable associations of key confounding variables listed in Fig. 1 to characterize their relationships with child outcomes. As shown in Table 2, older children (6–8 years) performed better on early language and numeracy tasks compared to the 4–5.9-year age group (β = 1.48, *p* < 0.0001 for early language, β = 4.49, p < 0.0001 for numeracy). In contrast, older children performed significantly worse on age-adjusted fluid intelligence (RCPM scores β = −19.42, p < 0.0001 for 6–8-year-old children compared to the 4–5.9-year age group), indicating that children in these settings are unable to develop their logical reasoning abilities at par with their chronological age. No significant sex differences were observed for any of the outcome measures. Children enrolled in private schools significantly outperformed those in government schools on numeracy tasks (β = 0.87, *p* < 0.001) and marginally on early language tasks (Table 2). The frequency of smartphone usage suggested an inverted U-shaped relationship with children's cognitive outcomes, such that none and the highest exposures were related to poorer cognitive outcomes compared to moderate smartphone usage (about once a week); although not statistically significant (Table 2).

**Table 2:** Univariable linear associations between child, caregiver, and household characteristics with child cognitive and academic outcomes adjusted for child age.

Variable	RCPM Percentile Rank, β (SE)^%^	*p* value	ASER Early language score, β (SE)	p value	ASER Numeracy score, β (SE)	p value
Range	0.1–99.9		1–9		0–14	
**Child characteristics**						
**Age (in years)** 4–5.9 6–8	Ref **-19.42 (1.95)**	< 0.001	Ref 1**.48 (0.09)**	< 0.001	Ref 4**.49 (0.19)**	< 0.001
**Sex** Boy Girl	Ref -1.78 (1.82)	0.32	Ref 0.12 (0.08)	0.12	Ref 0.26 (0.16)	0.11
**Type of School** Not enrolled Government Private	-1.52 (4.15) Ref -1.38 (2.44)	0.71 - 0.57	-0.31 (0.18) Ref 0.18 (0.11)	0.08 - 0.09	**-1.13 (0.36)** Ref **0.87 (0.22)**	0.002 - < 0.001
**Smartphone or tablet usage** Nil Rare Moderate Often	-1.54 (4.04) Ref 4.27 (3.27) -2.02 (2.83)	0.70 - 0.19 0.47	-0.31 (0.18) Ref 0.05 (0.14) -0.21 (0.12)	0.08 - 0.67 0.08	-0.28 (0.36) Ref 0.21 (0.29) -0.19 (0.26)	0.43 - 0.46 0.46
**Caregiver characteristics**						
**Mother's current age**	−0.33 (0.19)	0.09	0.004 (0.01)	0.59	0.03 (0.02)	0.10
**Mother's education** Illiterate or Primary Standard 5–8 Standard 9–12 Diploma/Graduate or above	Ref -2.79 (5.04) 3.28 (4.71) 3.00 (5.26)	- 0.58 0.48 0.57	Ref 0.19 (0.21) 0.38 (0.21) 0.37 (0.23)	- 0.37 0.06 0.11	Ref -0.15 (0.46) 0.53 (0.43) **0.97 (0.49)**	- 0.74 0.22 0.04
**Maternal IQ** RSPM score	**0.39 (0.16)**	0.01	**0.02 (0.007)**	0.01	**0.03 (0.01)**	0.04
**Maternal EPDS scores** EPDS score ≤ 13 EPDS score > 13	Ref -6.15 (3.25)	- 0.058	Ref -0.13 (0.14)	- 0.35	Ref **-0.82 (0.30)**	- 0.006
**Maternal social support** MSS score	-0.12 (0.09)	0.19	0.00 (0.00)	0.82	0.01 (0.01)	0.34
**Mother-child relationship** Low conflict + high closeness Low conflict + low closeness High conflict + high closeness High conflict + low closeness	Ref -2.29 (2.39) **5.41 (2.37)** 3.27 (2.39)	- 0.33 0.02 0.17	Ref **-0.23 (0.11)** -0.02 (0.11) -0.05 (0.11)	- 0.03 0.83 0.64	Ref -0.15 (0.23) 0.27 (0.23) 0.21 (0.23)	- 0.52 0.24 0.37
**Father's education** Illiterate or Primary Standard 5–8 Standard 9–12 Diploma/Graduate or above	Ref -0.05 (3.21) **7.32 (3.05)** **8.12 (3.69)**	- 0.98 0.01 0.02	Ref 0.03 (0.14) 0.19 (0.13) 0.27 (0.16)	- 0.79 0.14 0.09	Ref 0.53 (0.29) **0.95 (0.27)** **1.75 (0.33)**	- 0.07 0.004 < 0.001
**Household characteristics**						
**Number of children in the HH** 0 1 2 ≥ 3	0 -2.12 (3.11) -0.37 (3.17) -3.51 (3.68)	0.49 0.91 0.34	0 0.02 (0.15) -0.10 (0.15) -0.25 (0.17)	0.91 0.49 0.14	0 **-0.93 (0.30)** **-0.61 (0.30)** **-1.17 (0.35)**	0.002 0.04 0.0008

%Models with RCPM Percentile rank as the outcome were not adjusted for child age, since the outcome score is already age-standardized; Statistically significant associations (p < 0.05) are marked in bold; SE-standard error.

Higher levels of maternal education were associated with better cognitive and academic outcomes in children, particularly for numeracy tasks (β = 0.97, *p* = 0.04 for mothers with diploma/graduate education and above compared to mothers with up to primary level education). Higher paternal education (high school and above compared to primary school education) was also associated with significantly higher fluid intelligence and numeracy scores but not early language scores. Maternal IQ was significantly and positively associated with all cognitive and academic outcomes; every unit increase in maternal RSPM scores led to 0.39 units increase in RCPM percentile rank (*p* = 0.01), 0.02 units increase in early language scores (p = 0.01) and 0.03 units increase in numeracy scores (p = 0.04). Maternal depressive symptoms were associated with lower fluid intelligence (β = −6.15, *p* = 0.058), and significantly lower numeracy scores (β = −0.82, *p* = 0.006), but unrelated to early language scores. Maternal social support was not associated with any child outcome. Finally, compared to parent-child relationships, those with low conflict *and* low closeness (potentially indicative of low levels of caregiver-child engagement) were associated with poorer early language outcomes. On the other hand, high conflict *and* high closeness (potentially indicative of high levels of caregiver-child engagement) was associated with significantly better RCPM scores (β = 5.41, *p* = 0.02). Interestingly, presence of three or more children in the household was negatively associated with numeracy scores of the index child (β = −1.17, *p* = 0.0008, Table 2).

### Availability of books, play materials, and caregiver-child engagement in learning activities

#### Subscale 1: Household books

14 % (*n* = 132) children in our sample had no age-appropriate books in their household, or their caregivers were unaware of the number of books available (Supplementary table 1), while more than half the children (55.2 %, *n* = 519) had six or more books. Higher number of household books was significantly associated with children spending less time on smartphones or tablets to play videogames (χ2 = 66.46, *p* ≤0.0001). A total of 70 % of children with only 1–2 books used smartphones and tablets >3 days a week, compared to 44.9 % who had ≥6 age-appropriate books.

#### Subscale 2: Sources of play materials

Almost all children had store-bought toys (*n* = 912; 97 %), but also played with household objects (*n* = 859; 91.4 %), homemade toys (*n* = 754; 80.2 %), and outside objects (*n* = 727; 77.3 %). This is reflected in this subscale's high median score and low variability (median = 4, IQR = 1 out of a possible total score of 4) (Supplementary table 1).

#### Subscale 3: Variety of play materials

Toys for pretend play (95.5 %; Supplementary table 1) were the highest, while other varieties were also common: toys for drawing and writing (92.2 %), musical (78.1 %), stacking (76.5 %), or moving objects (87.8 %). In contrast, toys to teach shapes and colours were only available to 63.6 % children, and picture books to 48.2 %. This subscale showed more variation in scores, with the median being 6 (IQR 2.25) out of a possible total score of 7. Among children with the maximum variety of toys (7), only 35 % reported high levels of smartphone and tablet usage, compared to 55–76 % with fewer varieties of toys (1–6).

#### Subscale 4: Caregiver activities with the index child

The most common parent-child engagement strategy was playing with toys (81.6 %), followed by counting/drawing/naming objects (77.4 %), reading books to the child (77.2 %), and outdoor play (73.6 %). Fewer parents engaged in story telling (66.6 %) and singing songs (52.6 %). This subscale also demonstrated considerable variation, with the median score being 5 (IQR 3) out of a possible total score of 6. Similar to household books, higher number of caregiver-child engagements was also significantly associated with less time spent by the child on smartphones or tablets (χ2 = 119.1, *p* ≤0.0001) - compared to 43.3 % children whose parents reported engaging in 4–6 activities, >73 % of those whose parents engaged in 1–2 activities reported the highest frequency of smartphone usage.

### Associations between the home-learning environment and cognitive and academic outcomes in children

*Fluid intelligence (RCPM scores):* Availability of a higher number of household books compared to none showed a positive association with fluid intelligence in the fully adjusted model, particularly if ≥6 books were available (β = 0.25, *p* = 0.015; Table 3). Varieties of play materials were significantly associated with higher fluid intelligence across all models. The coefficient values remained consistent, with β = 0.06 (*p* = 0.007) in Model 1, β = 0.05 (*p* = 0.02) in Model 2, and β = 0.06 (p = 0.01) in Model 3, indicating minimal confounding by the selected variables. Caregiver activities with the index child also showed a strong and consistent positive association with fluid intelligence outcomes. The total FCI score demonstrated a significant association with RCPM percentile rank, with β = 0.04 (*p* < 0.0001) in Model 1, β = 0.04 (*p* = 0.0002) in Model 2, and β = 0.05 (p < 0.0001) in Model 3. These results highlight the positive impact of a rich home-learning environment on fluid intelligence outcomes (Table 3).

**Table 3:** Association of the home-learning environment with children's cognitive and academic outcomes adjusted for household, caregiver, and child characteristics.

Home-learning environment	Model 1^+^	P value	Model 2^++^	P value	Model 3^+++^	P value
**RCPM percentile rank**						
**Household books** None or don't know 1–2 3–5 ≥ 6	Ref 0.16 (0.13) 0.19 (0.12) **0.23 (0.10)**	- 0.20 0.10 0.03	Ref 0.20 (0.13) 0.23 (0.12) **0.23 (0.11)**	- 0.12 0.059 0.033	Ref 0.21 (0.13) **0.23 (0.12)** **0.25 (0.11)**	- 0.09 0.045 0.015
**Sources of play materials**	0.06 (0.05)	0.18	0.06 (0.05)	0.19	0.06 (0.05)	0.18
**Varieties of play materials**	**0.06 (0.02)**	0.007	**0.05 (0.02)**	0.02	**0.06 (0.02)**	0.01
**Caregiver activities with the index child**	**0.08 (0.02)**	< 0.0001	**0.08 (0.02)**	0.0001	**0.08 (0.02)**	< 0.0001
**Total FCI Score**	**0.04 (0.01)**	< 0.0001	**0.04 (0.01)**	0.0002	**0.05 (0.01)**	< 0.0001
**ASER early language score**						
**Household books** None or don't know 1–2 3–5 ≥ 6	Ref 0.07 (0.11) 0.18 (0.10) **0.27 (0.09)**	- 0.55 0.08 0.004	Ref 0.10 (0.11) 0.19 (0.10) **0.29 (0.10)**	- 0.38 0.08 0.002	Ref 0.12 (0.11) 0.19 (0.10) **0.29 (0.09)**	- 0.29 0.06 0.001
**Sources of play materials**	0.04 (0.04)	0.28	0.04 (0.04)	0.40	0.05 (0.04)	0.27
**Varieties of play materials**	0.03 (0.02)	0.11	0.03 (0.02)	0.14	0.04 (0.02)	0.07
**Caregiver activities with the index child**	**0.04 (0.02)**	0.006	**0.04 (0.02)**	0.02	**0.04 (0.02)**	0.01
**Total FCI Score**	**0.02 (0.01)**	0.01	**0.02 (0.01)**	0.02	**0.03 (0.01)**	0.009
**ASER numeracy score**						
**Household books** None or don't know 1–2 3–5 ≥ 6	Ref -0.04 (0.10) 0.09 (0.09) **0.21 (0.08)**	- 0.69 0.32 0.006	Ref -0.02 (0.10) 0.07 (0.09) **0.20 (0.08)**	- 0.87 0.41 0.009	Ref 0.03 (0.09) 0.04 (0.09) 0.10 (0.08)	- 0.76 0.64 0.19
**Sources of play materials**	0.03 (0.04)	0.46	0.01 (0.04)	0.69	0.02 (0.03)	0.62
**Varieties of play materials**	0.03 (0.02)	0.07	0.03 (0.02)	0.14	0.03 (0.02)	0.12
**Caregiver activities with the index child**	0.02 (0.01)	0.15	0.01 (0.01)	0.41	0.01 (0.01)	0.34
**Total FCI Score**	0.02 (0.008)	0.06	0.01 (0.009)	0.19	0.01 (0.008)	0.15

+Model 1: Child age, household characteristics (number of children in the household other than index child, SES)

++Model 2: Model 1 + maternal characteristics (maternal age, maternal IQ, depressive symptoms, social support, relationship with child- closeness/conflict)

+++Model 3: Model 2 + school attendance and type

Models with RCPM percentile rank as the outcome measure is not adjusted for child age as the outcome measure is already age-adjusted.

Statistically significant associations (p < 0.05) are marked in bold.

*Early language (ASER-early language scores):* A significant positive association was noted across all models for children with ≥6 household books and early language outcomes. Varieties of play materials were borderline associated with early language scores in Model 3, after adjusting for child age, type and school attendance. Significant positive associations were also found across all models for the number of caregiver activities and the total FCI score. When caregiver activity scores were categorized into tertiles, early language outcomes were significantly associated with only the highest activity category (six or more activities, see Supplementary Table 2). Therefore, gains in early language outcomes may only be possible through very high levels of caregiver engagement.

*Numeracy (ASER-numeracy scores):* Having ≥6 household books compared to none was also significantly associated with children's numeracy outcomes in models adjusted for household and maternal factors. However, the strength of this relationship reduced considerably when the model was further adjusted for the type of school children attended (Table 3). We observed a decreasing gradient in the availability of six or more books in children's households as a function of school attendance. While 62.5 % of children enrolled in private schools had ≥6 books at home, this number reduced to 41.8 % for those attending government schools, and 10.3 % for those yet to enrol in schools. In fact, 45 % of children yet to enrol in schools did not have any books at home (data not shown).

None of the other home-learning environment measures showed significant associations with numeracy outcomes across any of the models. For varieties of play materials, association with numeracy scores was marginally significant in Model 1, this association weakened in Models 2 and 3. Similarly, caregiver activities were not significantly associated with numeracy outcomes in any model. The total FCI score showed a trend towards significance in Model 1, but this effect became non-significant in Models 2 and 3. This suggests that the factors influencing numeracy outcomes may differ from those affecting fluid intelligence and early language outcomes.

## Discussion

Using data from a large sample of 940 mother-child dyads from an urban poor setting in South India, we found that having ≥6 age-appropriate books at home was a strong predictor of cognitive and early language outcomes during 4–8 years of age, particularly fluid intelligence and early language skills. Therefore, improving access to age-appropriate books in urban poor neighbourhoods could be a simple yet impactful intervention with multi-pronged benefits across diverse cognitive and academic outcomes in children.

Furthermore, while caregiver-child engagement activities and the overall home-learning environment were significantly associated with children's logical reasoning abilities and early language, numeracy skills were largely independent of the home-learning environment. Adjusting for school attendance and school type significantly diminished the association between number of household books and numeracy outcomes. It is possible that school-based learning in urban poor settings influences numeracy skills more than the home environment. We speculate that books available in urban poor households, or the dominant caregiver engagement activities such as playing with toys, reading to the child, and outdoor play may be geared towards promoting logical reasoning abilities and enhancing vocabulary and early language skills, with minimal content on numeracy skills. This relationship between poverty and poor numeracy skills has been demonstrated in other recent studies. For example, a study based in low-income settings of South Africa did not find any relationship between the home-learning environment and numeracy skills [23]. Similarly, a study based in high- versus low-SES households in Mexico demonstrated that home-learning activities promoting numeracy skills led to improvements in numeracy outcomes only in the high-SES, but not in the low-SES households [24]. Interestingly, this plays out at the country level as well, with low numeracy skills being most prevalent in the poorest populations within a country [25]. In India, numeracy skills often rely on textbook-based pedagogy [26]. In our sample, we found that children attending private schools were significantly more adept at numeracy skills compared to those attending government schools, or yet to enrol in schools. This highlights the multi-layered and complex nature of children's cognitive and academic development, and the critical need for not only a supportive caregiving environment, but also the parallel need for caregiver training on methods to improve numeracy skills through numeracy focused home-learning activities and access to high-quality schools.

The premise of our study is that child development is a socially and systemically determined phenomenon with influences ranging beyond the individual to the child's immediate family environment, its neighbourhood, and the wider social and economic environment into which children are born. This is also in line with Bronfenbrenner's ecological systems theory [27], as well as other socioecological approaches. Therefore, health and development according to these approaches, that we see at individual level, often arise through complex interactions between attributes at the micro-, meso-, and the macro-level ranging across caregiver engagement, parent-child relationships, caregiver well-being, access to quality education, and other public services all of which contribute to cognitive and academic skills. Hence our study aligns with long standing theories based on both human and animal studies, of the importance of providing a nurturing and resource-rich enriched environment to help children reach their full potential.

This study addresses two main limitations in the current literature. First, most studies are concentrated in the Global North, and second, they focus primarily on the early childhood period [28]. Our study examines the critical role that the home-learning environment continues to plays in shaping cognitive and academic outcomes cognitive outcomes of children in a low-and-middle-income country in the Global South context, and extends beyond the first three years from 4 to 8 years - critical for cognitive, language, and numeracy development. These foundational years mark the initiation and entry into formal learning, and crucially influence life-long academic and economic outcomes, and the quality of human capital formation. Furthermore, our study adds to the limited literature on child outcomes in urban poor neighbourhoods of large metropolises, a particularly vulnerable setting characterized by the presence of multiple risk factors including poor nutrition, unsafe or poor air quality, exposure to heavy metals and pesticides, limited access to quality education, exposure to violence at home or community settings, which cumulatively leave adverse imprints on children's survival and thriving [29].

Univariable analysis revealed that child-, caregiver-, and household-related factors known to influence child reasoning, early language, and numeracy outcomes during 0–3 years, continue to shape cognitive and academic abilities during 4–8 years in the expected directions. Higher levels of parental education were related to improved cognitive and academic outcomes across all examined domains as also noted in previous studies [30,31]. As demonstrated earlier [32], maternal IQ was found to be a better predictor of children's cognitive outcomes compared to maternal education. Although intelligence is a genotypic and heritable trait, gene-environment interactions in the form of the availability of cognitively stimulating environments in conjunction with genetic traits influence children's cognitive outcomes. Similarly, our study confirmed that concurrently measured maternal depressive symptoms are associated with poorer child cognitive and academic outcomes, even when children were between 4 and 8 years. This is in line with previous studies, that also showed that maternal depressive symptoms are linked to lower child cognitive scores [33,34]. Importantly, we did not observe sex differences in cognitive outcomes in this relatively young group of predominantly 4–6-year old children growing up in urban poor households in India. Longitudinal follow-up of this cohort is likely to unfold this gender differences as children grow up.

An interesting observation from examining the relationship between the three cognitive and academic outcomes revealed that fluid intelligence measures were poorly correlated with early language and numeracy outcomes, potentially highlighting the predominance of rote learning methods employed in urban poor settings to teach the latter skills. We observed a significant and concerning decline in age-standardized logical reasoning and problem-solving skills in older versus younger children (Standardized RCPM scores), emphasizing the need for home- and school-based interventions to build this important cognitive function throughout the early childhood period. An additional noteworthy finding was that while early language and numeracy outcomes were moderately correlated, their predictors were distinct. For example, caregiver-child engagement activities predicted early language, but not numeracy outcomes, which may be explained by the activities that predominantly promote early language, but not numeracy skills. The type of school children attended influenced numeracy skills far more than early language outcomes.

In this urban poor context, we noted that ∼75 % of the children attended private schools. Relatively high levels of parental education (>50 % completed high school) along with access to affordable low-fee private schools that cater to the lower-middle-income groups in these settings could potentially influence this decision. Parents prefer private schools as the curriculum is typically taught in the English, and many parents perceive them to be of higher quality compared to public schools [35,36].

India recently revised the National Education Policy (NEP) in 2020 [37], with one of the most significant modifications being an increased focus on pre-primary education to build foundational skills required for later academic achievement. These include fostering creativity, problem-solving, effective communication skills, critical and analytical ways of thinking, and recognizing that social and emotional well-being is an essential component of children's holistic development and learning outcomes. Importantly, NEP 2020 suggests that the home-learning environment and parental caregiving practices play a significant role in shaping the foundational skills deemed critical for academic success. Therefore, the implications of our findings are particularly relevant for designing interventions to promote holistic child development and to reduce the growing learning crisis in urban India, LMICs, and the Global South.

### Strengths and limitations

Our study has several strengths. To the best of our knowledge, this is the first study from India that utilized the Family Care Indicators (FCI) tool to assess the home-learning environment and its influence on children's cognitive and academic outcomes during the preschool to early primary years (4 to 8 years), when children undergo rapid neurobiological and skill development, thereby offering a novel perspective on child development in this setting. Second, we adjusted for a wide range of confounders, including crucial maternal factors such as depressive symptoms, social support, mother-child relationship, as well as child factors (type and school attendance), which are rarely accounted for in previous studies. Third, we measured several important child outcomes using validated, performance-based tools commonly used in India, including the ASER tool frequently utilized in annual education surveys in India, allowing consistency and comparability. Fourth, our study covers the critical preschool age range (4–8 years) and is directly relevant to the recently implemented national education policy in India. The age groups (4–5.9-years – two years prior to primary school entry, and 6–8 years - first two years of formal school) are critical learning periods in the context of the Indian education system, thereby enhancing the relevance of our findings. Interventions designed based on these results are likely to produce high returns on investment when implemented in the foundational years of learning, both in home and school contexts. Finally, both exposure and outcome measures showed high inter-rater reliability, ensuring consistency in data collection methods.

Limitations of this study include: maternal IQ had a considerable amount of missing data (11.5 %), therefore, to avoid losing data during analysis, we imputed missing data, assuming that the data were missing at random. Furthermore, although the FCI captures the availability of household books and play materials, frequency and duration of their use was not assessed. Additionally, Additionally, the FCI tool only enquires about caregiver engagement with family members over 15 years of age, but does not specifically collect data on engagement with siblings or other children in the family who could be important sources of learning for the index child. This is an important limitation of the tool, and therefore, of our study. We recognize that the COVID-19 pandemic likely affected both the quality of home-learning environment, and children's cognitive and academic outcomes. However, since the Family care indicators tool only considers caregiver-child engagement in the past three days, and measures availability of books & toys already present in the household, we were unable to assess the impact of COVID-19 on the exposure and outcomes of interest. Finally, any extracurricular activities or additional tutoring that the index child engaged in, which could influence their cognitive and academic outcomes was not included. We aim to address these by including these items in future data collection sweeps in the COINCIDE study to help provide a more comprehensive and precise understanding of the diverse roles of the home, school, and neighbourhood in shaping children's cognitive and academic development. Expanding research to include diverse contexts and populations will enhance the generalizability of the findings and help in designing targeted interventions to promote child development across the globe. Therefore, a similar study in rural settings would be valuable in understanding how these factors influence child outcomes across diverse Indian settings.

### Conclusion and implications

Our study provides robust evidence that a stimulating home-learning environment, enriched with books, diverse play materials, and active caregiver engagement, is significantly associated with improvements in specific cognitive and academic outcomes (fluid intelligence and early language, but not numeracy) in Indian children growing up in urban poor neighbourhoods during the 4–8 years age. The data underscore the importance of improving access to learning materials in homes and schools (particularly age-appropriate books), and educating caregivers on the importance of engaging in a variety of cognitively stimulating activities that are responsive and age-appropriate, to help reduce disparities in cognitive and academic outcomes in children growing up in disadvantaged settings. This is likely to have positive spin-off effects on education, economic, and health outcomes throughout their lives.

## CRediT authorship contribution statement

**Eunice Lobo:** Writing – review & editing, Writing – original draft, Supervision, Project administration, Methodology, Investigation, Conceptualization. **Debarati Mukherjee:** Writing – review & editing, Validation, Supervision, Methodology, Investigation, Funding acquisition, Formal analysis, Conceptualization. **Pradeep Kumar Choudhury:** Writing – review & editing, Formal analysis, Conceptualization. **Giridhara Rathnaiah Babu:** Writing – review & editing, Project administration, Funding acquisition, Formal analysis, Conceptualization. **Prashanth Nuggehalli Srinivas:** Writing – review & editing, Funding acquisition. **Onno C.P. van Schayck:** Writing – review & editing.

## Funding

This work was supported by the DBT/10.13039/100010269Wellcome Trust 10.13039/501100009053India Alliance Team Science Grant [Grant No. IA/TSG/20/1/600023]. Prashanth Nuggehalli Srinivas was also supported by the DBT/10.13039/100010269Wellcome Trust 10.13039/501100009053India Alliance CRC Grant [Grant IA/CRC/20/1/600007]. Debarati Mukherjee was also supported by the 10.13039/100000061Fogarty International Center of the 10.13039/100000002National Institutes of Health under Award Number D43 TW011404. The funders had no role in study design, data collection and analysis, decision to publish, or manuscript preparation.

## Declaration of competing interest

The authors declare that they have no known competing financial interests or personal relationships that could have appeared to influence the work reported in this paper.

## Data Availability

Decisions regarding data sharing will be made by the COINCIDE consortium/publication committee on a case-by-case basis.

## Appendix A Supplementary data

Supplementary material 1 Image 1

Supplementary material 2 Image 2

## References

[1] Black M.M., Walker S.P., Fernald L.C.H., Andersen C.T., DiGirolamo A.M., Lu C. (2017). Early childhood development coming of age: science through the life course. Lancet.

[2] Lu C., Black M.M., Richter L.M. (2016). Risk of poor development in young children in low-income and middle-income countries: an estimation and analysis at the global, regional, and country level. Lancet Glob Health.

[3] World Health Organization (2018). Nurturing care for early childhood development: a framework for helping children survive and thrive to transform health and human potential. https://www.who.int/teams/maternal-newborn-child-adolescent-health-and-ageing/child-health/nurturing-care.

[4] Yang Q., Yang J., Zheng L., Song W., Yi L.J. (2021). Impact of home parenting environment on cognitive and psychomotor development in children under 5 years old: a meta-analysis. Front Pediatr.

[5] Yousafzai A.K., Obradović J., Rasheed M.A., Rizvi A., Portilla X.A., Tirado-Strayer N. (2016). Effects of responsive stimulation and nutrition interventions on children’s development and growth at age 4 years in a disadvantaged population in Pakistan: a longitudinal follow-up of a cluster-randomised factorial effectiveness trial. Lancet Glob Health.

[6] Roopnarine J.L., Dede Yildirim E. (2018). Paternal and maternal engagement in play, story telling, and reading in five Caribbean countries: associations with preschoolers’ literacy skills. Play.

[7] Aram D., Shapira R. (2012). Parent-child shared book reading and children’s language, literacy, and empathy development. Rivista italiana di educazione familiare.

[8] Dag N.C., Turkkan E., Kacar A., Dag H. (2021). Children’s only profession: playing with toys. North Clin Istanb.

[9] Rosa W. (2017). https://sdgs.un.org/goals/goal4.

[10] Hoff E. (2003). The specificity of environmental influence: socioeconomic status affects early vocabulary development via maternal speech. Child Dev.

[11] Linver M.R., Brooks-Gunn J., Kohen D.E. (2002). Family processes as pathways from income to young children’s development. Dev Psychol.

[12] Bornstein M.H., Putnick D.L. (2012). Cognitive and socioemotional caregiving in developing countries. Child Dev.

[13] Hentschel E., Tomlinson H., Hasan A., Yousafzai A., Ansari A., Tahir-Chowdhry M. (2024). Risks to child development and school readiness among children under six in Pakistan: findings from a nationally representative phone survey. Int J Early Childhood.

[14] Nampijja M., Kizindo R., Apule B., Lule S., Muhangi L., Titman A. (2018). The role of the home environment in neurocognitive development of children living in extreme poverty and with frequent illnesses: a cross-sectional study. Wellcome Open Res.

[15] Lobo E., Deepa R., Mandal S., Menon J.S., Roy A., Dixit S. (2024). Protocol of the nutritional, psychosocial, and environmental determinants of neurodevelopment and child mental health (COINCIDE) study. Wellcome Open Res.

[16] Babu G.R., Murthy G.V.S., Deepa R., Yamuna Prafulla, Kumar H.K. (2016). Maternal antecedents of adiposity and studying the transgenerational role of hyperglycemia and insulin (MAASTHI): a prospective cohort study. BMC Pregnancy Childbirth.

[17] Lobo E., Ana Y., Deepa R., Shriyan P., Sindhu N.D., Karthik M. (2022). Cohort profile: maternal antecedents of adiposity and studying the transgenerational role of hyperglycaemia and insulin (MAASTHI). BMJ Open.

[18] Harris P.A., Taylor R., Thielke R., Payne J., Gonzalez N., Conde J.G. (2009). Research electronic data capture (REDCap)--a metadata-driven methodology and workflow process for providing translational research informatics support. J Biomed Inform.

[19] Hamadani J.D., Tofail F., Hilaly A., Huda S.N., Engle P., Grantham-McGregor S.M. (2010). Use of family care indicators and their relationship with child development in Bangladesh. J Health Popul Nutr.

[20] Bhogle S., Prakash I.J. (1992). Performance of Indian children on the coloured progressive matrices. Psychol Stud.

[21] Vagh S.B. (2012). https://img.asercentre.org/docs/Aser%20survey/Tools%20validating_the_aser_testing_tools__oct_2012__3.pdf.

[22] Fernandes M.C., Srinivasan K., Stein A.L., Menezes G., Sumithra R., Ramchandani P.G. (2011). Assessing prenatal depression in the rural developing world: a comparison of two screening measures. Arch Womens Ment Health.

[23] Merkley R., Sernoskie E., Cook C.J., Howard S.J., Makaula H., Mshudulu M. (2023). “We Don’t have things for counting”: an exploration of early numeracy skills and home learning experiences of children growing up in poverty in South Africa. J Numer Cognit.

[24] Susperreguy M.I., Jiménez Lira C., Xu C., LeFevre J.-A., Blanco Vega H., Benavides Pando E.V. (2021). Home learning environments of children in Mexico in relation to socioeconomic status. Front Psychol.

[25] Bruine de Bruin W., Slovic P. (2021). Low numeracy is associated with poor financial well-being around the world. PloS One.

[26] Chaurasia P. (2020). Mathematics Education in India: Retrospectives and Perspectives Indian. J Adult Edu.

[27] Bronfenbrenner U. (1989).

[28] Han J., Cui N., Lyu P., Li Y. (2023). Early-life home environment and child cognitive function: a meta-analysis. Personal Individ Differ.

[29] Ambey R., Gaur A., Gupta R., Patel G. (2013). Urban poor children. Australas Med J.

[30] Cermakova P., Chlapečka A., Csajbók Z., Andrýsková L., Brázdil M., Marečková K. (2023 Feb 16). Parental education, cognition and functional connectivity of the salience network. Sci Rep.

[31] Dickson M., Gregg P., Robinson H. (2016 Oct 1). Early, late or never? When does parental education impact child outcomes?. Econ J.

[32] Lean R.E., Paul R.A., Smyser C.D., Rogers C.E. (2018). Maternal intelligence quotient (IQ) predicts IQ and language in very preterm children at age 5 years. J Child Psychol Psychiatry.

[33] Rogers A., Obst S., Teague S.J., Rossen L., Spry E.A., Macdonald J.A. (2020 Nov 1). Association between maternal perinatal depression and anxiety and child and adolescent development: a Meta-analysis. JAMA Pediatr.

[34] Lautarescu A., Craig M.C., Glover V. (2020). Prenatal stress: effects on fetal and child brain development. Int Rev Neurobiol.

[35] Okitsu T., Edwards D.B., Mwanza P., Miller S. (2023). Low-fee private preschools as the symbol of imagined ‘modernity’? – parental perspectives on early childhood care and education (ECCE) in an urban informal settlement in Zambia. Int J Educ Dev.

[36] Härmä J. (2011). Low cost private schooling in India: is it pro poor and equitable?. Int J Edu Dev.

[37] Ministry of Human Resources Development. Government of India. National Education Policy 2020. Available at: https://www.education.gov.in/sites/upload_files/mhrd/files/NEP_Final_English_0.pdf.

