# Supplementary material for: Home-learning environment and cognitive and academic outcomes among children aged 4–8 years: A cross-sectional study from South India
Source: Dialogues Health. 2025 Sep 6;7:100238. doi: 10.1016/j.dialog.2025.100238 (PMC12455069; doi:10.1016/j.dialog.2025.100238)
Supplement: Supplementary file 1 — Supplementary material 1 [file mmc1.docx]

**Supplementary files**

# **Supplementary checklist 1: Strengthening the Reporting of Observational Studies in Epidemiology (STROBE) statement checklist**

STROBE Statement—Checklist of items that should be included in reports of ***cross-sectional studies***

|  | Item No | Recommendation | Page No |
| --- | --- | --- | --- |
| **Title and abstract** | 1 | (*a*) Indicate the study’s design with a commonly used term in the title or the abstract | 1 (Title page) |
|  |  | (*b*) Provide in the abstract an informative and balanced summary of what was done and what was found | 1 |
| Introduction | | | |
| Background/rationale | 2 | Explain the scientific background and rationale for the investigation being reported | 2-3 |
| Objectives | 3 | State specific objectives, including any pre-specified hypotheses | 3 |
| Methods | | | |
| Study design | 4 | Present key elements of study design early in the paper | 4 |
| Setting | 5 | Describe the setting, locations, and relevant dates, including periods of recruitment, exposure, follow-up, and data collection | 4 |
| Participants | 6 | (*a*) Give the eligibility criteria, and the sources and methods of selection of participants | 4 |
| Variables | 7 | Clearly define all outcomes, exposures, predictors, potential confounders, and effect modifiers. Give diagnostic criteria, if applicable | 5-6  Supplementary files pages 4-6 |
| Data sources/ measurement | 8* | For each variable of interest, give sources of data and details of methods of assessment (measurement). Describe comparability of assessment methods if there is more than one group | 5-6  Supplementary files pages 4-6 |
| Bias | 9 | Describe any efforts to address potential sources of bias | Supplementary files pages 5-6 |
| Study size | 10 | Explain how the study size was arrived at | 4 |
| Quantitative variables | 11 | Explain how quantitative variables were handled in the analyses. If applicable, describe which groupings were chosen and why | 5-6  Supplementary files pages 4 to 6 |
| Statistical methods | 12 | (*a*) Describe all statistical methods, including those used to control for confounding | 5-6  Supplementary files pages 5-6 |
|  |  | (*b*) Describe any methods used to examine subgroups and interactions | Not applicable |
|  |  | (*c*) Explain how missing data were addressed | Supplementary files page 6 |
|  |  | (*d*) If applicable, describe analytical methods taking account of sampling strategy | Not applicable |
|  |  | (*e*) Describe any sensitivity analyses | Not applicable |
| Results | | | |
| Participants | 13* | (a) Report numbers of individuals at each stage of study—eg numbers potentially eligible, examined for eligibility, confirmed eligible, included in the study, completing follow-up, and analysed | 4  All eligible participants were analysed in this study |
|  |  | (b) Give reasons for non-participation at each stage | Not applicable |
|  |  | (c) Consider use of a flow diagram | Not applicable |
| Descriptive data | 14* | (a) Give characteristics of study participants (eg demographic, clinical, social) and information on exposures and potential confounders | 8  Table 1 |
|  |  | (b) Indicate number of participants with missing data for each variable of interest | Table 1 |
| Outcome data | 15* | Report numbers of outcome events or summary measures | 8-17 |
| Main results | 16 | (*a*) Give unadjusted estimates and, if applicable, confounder-adjusted estimates and their precision (eg, 95% confidence interval). Make clear which confounders were adjusted for and why they were included | 5-6  Supplementary files pages 5-6 |
|  |  | (*b*) Report category boundaries when continuous variables were categorized | Table 2 |
|  |  | (*c*) If relevant, consider translating estimates of relative risk into absolute risk for a meaningful time period | Not applicable |
| Other analyses | 17 | Report other analyses done—eg analyses of subgroups and interactions, and sensitivity analyses | Not applicable |
| Discussion | | | |
| Key results | 18 | Summarise key results with reference to study objectives | 18-19 |
| Limitations | 19 | Discuss limitations of the study, taking into account sources of potential bias or imprecision. Discuss both direction and magnitude of any potential bias | 21-22 |
| Interpretation | 20 | Give a cautious overall interpretation of results considering objectives, limitations, multiplicity of analyses, results from similar studies, and other relevant evidence | 18-22 |
| Generalisability | 21 | Discuss the generalisability (external validity) of the study results | 18-22 |
| Other information | | | |
| Funding | 22 | Give the source of funding and the role of the funders for the present study and, if applicable, for the original study on which the present article is based | 1 (Title page) |

*Give information separately for exposed and unexposed groups.

**Note:** An Explanation and Elaboration article discusses each checklist item and gives methodological background and published examples of transparent reporting. The STROBE checklist is best used in conjunction with this article (freely available on the Web sites of PLoS Medicine at http://www.plosmedicine.org/, Annals of Internal Medicine at http://www.annals.org/, and Epidemiology at http://www.epidem.com/). Information on the STROBE Initiative is available at www.strobe-statement.org.

# **Supplementary Data 1: Additional details on study methods**

***Exposure assessment***

The Family Care Indicators (FCI) tool was used to collect data on play and learning activities, including reading books, storytelling, singing, outdoor play, counting, naming, and drawing objects, conducted over the past three days. The tool has been validated in the South Asian and African contexts in children aged 18-months to 7 years [1, 2], to assess the home-learning environment through specific domain scores, which are subsequently added to provide a total score representative of the overall home environment. Each domain score is derived from summing positive responses to individual items. This allowed us to conduct a detailed evaluation of the associations between the individual domains, as well as the holistic home-learning environment, and children’s cognitive outcomes.

***Outcomes assessment***

Fluid intelligence was measured using the Raven’s Coloured Progressive Matrices (RCPM) [3]. The test comprises three sets of twelve items, each increasing in difficulty. In the first set (Set A), the child has to correctly identify the piece that is missing in an image to complete a geometrical pattern. Six response options are provided, of which only one is correct. In the next two sets (Set AB and Set B), the child has to complete a four-piece geometrical pattern in which one piece is missing. Again, six response options are available to the child from which they have to choose the correct option. Each correct response is scored as ‘1’, while incorrect responses receive a score of ‘0’. The RCPM total raw score was calculated by summing across all correct responses (therefore scores ranged from 0-36 across all 3 sets). Raw scores were converted to scaled scores (mean = 100, standard deviation = 15) and percentile ranks using a norm table that standardizes RCPM scores across age groups as per Indian norms. Trained assessors administered the test following standard operating procedures prescribed in the RCPM manual.

To test early language and numeracy skills, we used the preschool version of the Annual Status of Education Report (ASER) tool, a standardized and validated tool to assess early language and numeracy skills in 4-8-year-old children in India [4].

Early language was assessed through picture descriptions [5], early reading skills such as naming English alphabets (upper and lower-case), reading words, and one simple paragraph, and reading and listening comprehension (assessed by asking the child questions related to the story they read, or one that was read to them). Children progressed to the next item only if they met criteria for successfully completing previous skills (e.g. tested on word reading only if they were able to name at least 5 out of 8 letters).

Numeracy skills were also assessed in English through items that required children to count objects, complete oral word problems, recognize single- and double-digit numbers and identify which of them were larger versus smaller in value. The test also included addition and subtraction of single- and double-digit numbers. As in the early language test, higher-level skills were assessed only when children passed the criteria in previous items.

Responses were coded as “Correct”, “Incorrect” or “Don’t know”, with correct responses assigned a score of ‘1’. “Incorrect” or “Don’t know” responses received a score of 0. In this study, possible scores on the early language test ranged from 0-9 and for the early numeracy test, it ranged from 0-14.

***Confounding variables***

Based on the available literature, regression models were adjusted for the following confounders:

*Child factors* included child age, school attendance and type (none versus private versus public).

*Maternal factors* included maternal age, IQ (measured using the Raven’s Standard Progressive Matrices [6] - a non-verbal intelligence test similar to the RCPM, consisting of 50 items of increasing difficulty; raw scores were used). To assess maternal depressive symptoms, we used the Edinburgh Postnatal Depression Scale (EPDS). An EPDS score cut-off of ≥13 was used to categorize mothers as having depressive symptoms, as validated previously in our settings [5]. Maternal social support was measured using a tool developed by St. John’s Research Institute, India. The tool assesses four types of social support: emotional, instrumental, informational, and appraisal [7]. To assess mother-child relationship, we used the child-parent relationship scale (CPRS) [8]. This provides two component scores for conflict and closeness. These variables were categorized as high or low using the median cut-off (see Table 1). We generated a new variable comprising the following four levels as the confounding variable representing maternal-child relationship: (i) low conflict-high closeness; (ii) low conflict-low closeness; (iii) high conflict-low closeness; (iv) high conflict-high closeness. The low-conflict/high-closeness group served as the reference level.

*Household-level factors* included socioeconomic status (SES), and the number of children in the household including the index child. Socioeconomic status (SES) was calculated using Principal Component Analysis score derived using the following variables: maternal and paternal education and occupation, crowding (number of household members divided by the number of rooms used for sleeping), source of drinking water (piped into household vs other), material of the walls, floor and roof, toilet facility (owned versus shared), household asset list (Electricity, Mattress, Pressure cooker, Chair, Cot or Bed, Table, Electrical fan, Radio or Transistor, Black & White Television, Colour Television, Sewing Machine, Mobile Telephone, Landline Telephone, Internet, Computer or tablet, Refrigerator, Air conditioner / Cooler, Washing Machine, Watch or Clock, Bicycle, Motorcycle or Scooter, Auto or Tempo, Car, Water-pump), ownership by any household member of real estate, bank account, or a government-authorized card identifying the family as living below the poverty line, and presence of a domestic servant in the house. The first six components (PC1-PC6), which explained 77.7% of the variance, were included in our models to adjust for SES

***Missing data and imputation***

Patterns of missing data are reported in Table 1. All but two variables (maternal IQ and frequency of smartphone usage) had <2% missing data. The highest proportion of missing data was in maternal IQ, measured using the Raven’s Standard Progressive Matrices (11.5%). The RSPM test was typically conducted at the end of the comprehensive maternal assessment (lasting approximately 90 to 120 minutes). Consequently, mothers often declined to participate in an additional assessment. Data were assumed to be missing at random.

Since maternal IQ is an important confounding variable in the proposed conceptual framework (Figure. 1), missing values were imputed 30 times using the ‘mice’ package in R prior to running each model, which imputes missing data according to the Multivariate Imputation by Chained Equation method. The summary of the regression results for each of the thirty imputed datasets were pooled using built-in functions in the ‘mice’ package, and reported in the results tables as regression coefficients progressively adjusted for household, maternal and child factors along with the p-value. A two-tailed p-value of <0.05 was considered to be statistically significant.

**References:**

1. Kariger P, Frongillo EA, Engle P, Britto PM, Sywulka SM, Menon P. Indicators of family care for development for use in multicountry surveys. *J Health Popul Nutr* 2012; 30(4): 472-86. doi: 10.3329/jhpn.v30i4.13417
2. Hamadani JD, Tofail F, Hilaly A, Huda SN, Engle P, Grantham-McGregor SM. Use of family care indicators and their relationship with child development in Bangladesh. *J Health Popul Nutr* 2010; 28(1): 23-33. doi: 10.3329/jhpn.v28i1.4520
3. Raven JC. Coloured Progressive Matrices, Sets A, A_B, B. *HK Lewis* 1962.
4. Vagh SB. Validating the ASER testing tools: Comparisons with reading fluency measures and the Read India measures. *Unpublished report Retrieved July* 2012; 30: 2018.Available at: https://img.asercentre.org/docs/Aser%20survey/Tools%20validating_the_aser_testing_tools__oct_2012__3.pdf
5. Fernandes MC, Srinivasan K, Stein AL, Menezes G, Sumithra R, Ramchandani PG. Assessing prenatal depression in the rural developing world: a comparison of two screening measures. *Arch Womens Ment Health* 2011; 14(3): 209-16.doi: 10.1007/s00737-010-0190-2
6. Raven JC. Raven standard progressive matrices. *Journal of Cognition and Development* 1938.
7. Anand SS, Vasudevan A, Gupta M, et al. Rationale and design of South Asian Birth Cohort (START): a Canada-India collaborative study. *BMC Public Health* 2013; 13(1): 79. doi: 10.1186/1471-2458-13-79
8. Pianta RC. Parent-child relationship scale. Charlottesville, VA: University of Virginia. 1992.

# **Supplementary Figure 1: Directed acyclic graph of home-learning environment and child outcomes, adjusting for confounders**


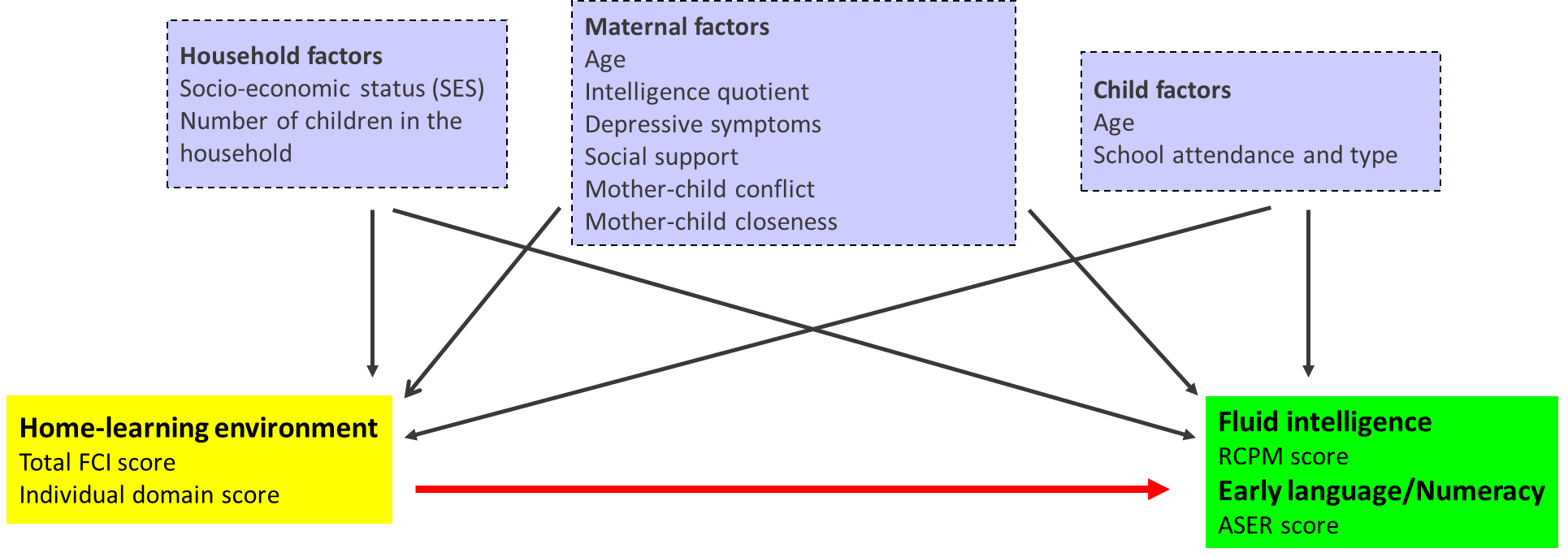


# **Supplementary Table 1: Characteristics of MAASTHI cohort participants (followed up during COINCIDE versus not followed up)**

| **Variables** | **COINCIDE participants**  **(N=940)** | **MAASTHI participants not followed up in COINCIDE (N=2022)** |
| --- | --- | --- |
| ***Mother’s age at interview (in years)***  < 20  20-30  31-40  41-50  ***Mean (SD)*** | 50 (5.3%)  794(84.5%)  93(9.9%)  3(0.3%)  24.4 (4.1) | 98 (4.9%)  1715 (84.8%)  206 (10.2%)  3 (0.1%)  24.3 (4) |
| ***Mother’s age at delivery (in years)***  *<20*  *20-30*  *31-40*  *41-50*  ***Mean (SD)*** | 34 (3.6%)  803(85.4%)  96 (10.2%)  3 (0.3)  24.6 (4.1) | 69(3.41%)  1653(81.8%  217 910.7%)  3 (0.1%)  24.6 (4) |
| ***Religion***  *Hindu*  *Christian*  *Islam*  *Not available* | 471 (50.1%)  22 (2.3%)  443 (47.1%)  1 (0.1%) | 1021 (50.5%)  86 (4.3%)  909 (45%)  2 ( 0.1%) |
| ***Mother’s education***  *Illiterate*  *Primary school*  *Middle school*  *High school*  *PUC / diploma*  *Graduate*  *Post-graduate* | 31 (3.3%)  30 (3.1%)  126 (13.4%)  430 (45.7%)  225 (23.9%)  84 (8.9%)  11 (1.1%) | 71 (3.5%)  129 (6.4%)  289 (14.3%)  868 (42.9%)  428 (21.2 %)  214 (10.6%)  20 ( 0.9%) |
| ***Father’s education***  *Illiterate*  *Primary school*  *Middle school*  *High school*  *PUC / diploma*  *Graduate*  *Post-graduate* | 74 (7.9%)  80 (8.5%)  149 (15.9%)  390 (41.5%)  159 (16.9%)  71 (7.6%)  13 (1.4%) | 195 (9.6%)  186 (9.2%)  339 (16.8%)  786 (38.9%)  336 (16.6%)  154 (7.6%)  18 (0.9%) |
| ***Mother’s occupation***  *Unemployed/Homemaker*  *Unskilled*  *Semiskilled*  *Skilled*  *Professional* | 887 (94.4%)  26 (2.8%)  8(0.8%)  15 (1.6%)  1(0.1%) | 1869 (92.4%)  82 (4.1%)  31(1.5%)  35 (1.7%)  2 (0.1%) |
| ***Father’s occupation***  *Unemployed*  *Unskilled*  *Semiskilled*  *Skilled*  *Clerical*  *Semi professional*  *Professional* | 3 (0.3%)  453 (48.2%)  280 (29.8%)  165 (17.6%)  9 (0.9%)  16 (1.7%)  11 (1.17%) | 10 (0.5 %)  987 (48.8%)  607 (30%)  332 (16.4%)  26 (1.3%)  23 (1.1%)  34 ( 1.7%) |
| ***Socio-economic status***  *Lower class*  *Upper lower class*  *Lower middle class*  *Upper middle class*  *Upper class* | 1(0.1%)  547 (58.2%)  291(31%)  93 (9.9%)  5 (0.5%) | 1(0.04%)  1116 (55.2%)  657 (32.5%)  236 (11.7%)  9 ( 0.4%) |

# **Supplementary Table 2: Family care indicators (FCI) among children in the MAASTHI cohort**

| **Subscale** | **Frequency n (%)** |
| --- | --- |
| ***Subscale 1 (Household books)*** | |
| None or don’t know  1-2  3-5  ≥6 | 132 (14.0%)  114 (12.1%)  175 (18.6%)  519 (55.2%) |
| ***Subscale 2 (Source of play materials)*** | |
| Homemade toys  Store-bought toys  Household objects  Outside objects  **Median (IQR)** | 754 (80.2%)  912 (97.0%)  859 (91.4%)  727 (77.3%)  **4 (1) (out of a possible 4)** |
| ***Subscale 3 (Variety of play materials)*** | |
| Musical objects  Drawing and writing  Picture books  Stacking toys  Moving objects  Shapes and colours  Pretend play  **Median (IQR)** | 734 (78.1%)  867 (92.2%)  453 (48.2%)  719 (76.5%)  825 (87.8%)  598 (63.6%)  898 (95.5%)  **6 (2.25) (out of a possible 7)** |
| ***Subscale 4 (Caregiver activities with the index child)*** | |
| Book reading  Storytelling  Singing songs  Outdoor play  Played with child with toys  Counting /drawing/ naming objects  **Median (IQR)** | 726 (77.2%)  626 (66.6%)  494 (52.6%)  692 (73.6%)  767 (81.6%)  728 (77.4%)  **5 (3) (out of a possible 6)** |

# **Supplementary Table 3: Association of the home-learning environment on children’s cognitive, early language, and numeracy outcomes**

| Home-learning environment | Model 1^+^ | P value | Model 2^++^ | P value | Model 3^+++^ | P value |
| --- | --- | --- | --- | --- | --- | --- |
| **RCPM percentile rank** | | | | | | |
| **Household books**  None or don’t know  1-2  3-5  ≥6 | Ref  0.16 (0.13)  0.19 (0.12)  **0.23 (0.10)** | -  0.20  0.10  0.03 | Ref  0.20 (0.13)  0.23 (0.12)  **0.23 (0.11)** | -  0.12  0.059  0.033 | Ref  0.21 (0.13)  **0.25 (0.12)**  **0.28 (0.11)** | -  0.11  0.04  0.009 |
| **Sources of play materials**  Score: 0-2 (N = 97)  Score: 3 (N = 269)  Score: 4 (N = 567) | Ref  -0.03 (0.12)  0.10 (0.11) | -  0.78  0.41 | Ref  -0.02 (0.12)  0.10 (0.11) | -  0.88  0.36 | Ref  -0.02 (0.12)  0.09 (0.12) | -  0.84  0.43 |
| **Varieties of play materials**  Score: 0-4 (N = 233)  Score: 5-6 (N = 415)  Score: 7 (N = 284) | Ref  **0.35 (0.08)**  **0.19 (0.09)** | -  <0.0001  0.04 | Ref  **0.32 (0.08)**  0.17 (0.09) | -  0.0002  0.085 | Ref  **0.33 (0.09)**  **0.19 (0.10)** | -  0.0001  0.049 |
| **Caregiver activities with child**  Score: 0-3 (N = 281)  Score: 4-5 (N = 262)  Score: 6 (N = 383) | Ref  **0.28 (0.09)**  **0.33 (0.08)** | -  0.001  <0.0001 | Ref  **0.27 (0.09)**  **0.30 (0.09)** | -  0.002  0.0006 | Ref  **0.29 (0.09)**  **0.30 (0.09)** | -  0.0009  0.0003 |
| **Total FCI Score**  Score: 0-11 (N = 255)  Score: 12-15 (N = 384)  Score: 16-17 (N = 280) | Ref  **0.32 (0.08)**  **0.32 (0.09)** | -  0.0001  0.0007 | Ref  **0.31 (0.08)**  **0.29 (0.10)** | -  0.0002  0.004 | Ref  **0.32 (0.08)**  **0.29 (0.10)** | -  0.0001  0.002 |
| **ASER early language score** | | | | | | |
| **Household books**  None or don’t know  1-2  3-5  ≥6 | Ref  0.07 (0.11)  0.18 (0.10)  **0.27 (0.09)** | -  0.55  0.08  0.004 | Ref  0.10 (0.11)  0.19 (0.10)  **0.29 (0.10)** | -  0.38  0.08  0.002 | Ref  0.12 (0.11)  0.19 (0.10)  **0.29 (0.09)** | -  0.30  0.06  0.001 |
| **Sources of play materials**  Score: 0-2 (N = 97)  Score: 3 (N = 269)  Score: 4 (N = 567) | Ref  0.11 (0.10)  0.13 (0.10) | -  0.29  0.21 | Ref  0.12 (0.10)  0.11 (0.10) | -  0.25  0.25 | Ref  0.13 (0.11)  0.14 (0.10) | -  0.22  0.17 |
| **Varieties of play materials**  Score: 0-4 (N = 233)  Score: 5-6 (N = 415)  Score: 7 (N = 284) | Ref  **0.15 (0.07)**  0.10 (0.08) | -  0.04  0.25 | Ref  0.14 (0.07)  0.09 (0.09) | -  0.06  0.32 | Ref  0.13 (0.07)  0.13 (0.09) | -  0.06  0.12 |
| **Caregiver activities with the index child**  Score: 0-3 (N = 281)  Score: 4-5 (N = 262)  Score: 6 (N = 383) | Ref  0.08 (0.07)  **0.21 (0.07)** | -  0.28  0.005 | Ref  0.05 (0.08)  **0.18 (0.08)** | -  0.49  0.01 | Ref  0.04 (0.08)  **0.20 (0.07)** | -  0.55  0.006 |
| **Total FCI Score**  Score: 0-11 (N = 255)  Score: 12-15 (N = 384)  Score: 16-17 (N = 280) | Ref  **0.16 (0.07)**  **0.20 (0.08)** | -  0.02  0.01 | Ref  0.14 (0.07)  0.17 (0.09) | -  0.053  0.058 | Ref  **0.15 (0.07)**  **0.22 (0.09)** | -  0.04  0.009 |
| **ASER numeracy score** | | | | | | |
| **Household books**  None or don’t know  1-2  3-5  ≥6 | Ref  -0.04 (0.10)  0.09 (0.09)  **0.21 (0.08)** | -  0.69  0.32  0.006 | Ref  -0.02 (0.10)  0.07 (0.09)  **0.20 (0.08)** | -  0.87  0.41  0.009 | Ref  -0.03 (0.09)  0.04 (0.09)  0.10 (0.08) | -  0.76  0.64  0.20 |
| **Sources of play materials**  Score: 0-2 (N = 97)  Score: 3 (N = 269)  Score: 4 (N = 567) | Ref  -0.02 (0.09)  0.02 (0.09) | -  0.77  0.82 | Ref  -0.03 (0.09)  0.00 (0.09) | -  0.78  0.99 | Ref  -0.04 (0.09)  -0.00 (0.09) | -  0.67  0.99 |
| **Varieties of play materials**  Score: 0-4 (N = 233)  Score: 5-6 (N = 415)  Score: 7 (N = 284) | Ref  **0.14 (0.06)**  0.09 (0.07) | -  0.03  0.22 | Ref  0.12 (0.07)  0.07 (0.07) | -  0.06  0.34 | Ref  **0.13 (0.06)**  0.08 (0.08) | -  0.03  0.26 |
| **Caregiver activities with child**  Score: 0-3 (N = 281)  Score: 4-5 (N = 262)  Score: 6 (N = 383) | Ref  -0.06 (0.06)  0.10 (0.06) | -  0.36  0.11 | Ref  -0.09 (0.06)  0.06 (0.06) | -  0.19  0.31 | Ref  -0.08 (0.06)  0.07 (0.06) | -  0.20  0.21 |
| **Total FCI Score**  Score: 0-11 (N = 255)  Score: 12-15 (N = 384)  Score: 16-17 (N = 280) | Ref  0.03 (0.06)  0.06 (0.07) | -  0.56  0.34 | Ref  0.01 (0.06)  0.02 (0.07) | -  0.89  0.80 | Ref  0.003 (0.06)  0.03 (0.07) | -  0.95  0.68 |

*^+Model 1: Child age, household characteristics (number of children in the household other than index child, SES)^*

*^++Model 2: Model 1 + maternal characteristics (maternal age, maternal IQ, depressive symptoms, social support, relationship with child- closeness/conflict)^*

*^+++Model 3: Model 2 + type of school attendance^*

*^Models with RCPM percentile rank as the outcome measure is not adjusted for child age as the outcome measure is already age-adjusted.^*

*^Statistically significant associations (p<0.05) are marked in bold.^*
