# Supplementary material for: Home-learning environment and cognitive and academic outcomes among children aged 4–8 years: A cross-sectional study from South India
Source: Dialogues Health. 2025 Sep 6;7:100238. doi: 10.1016/j.dialog.2025.100238 (PMC12455069; doi:10.1016/j.dialog.2025.100238)
Supplement: Supplementary file 2 — Supplementary material 2 [file mmc2.docx]

- **Supplementary checklist 1: Strengthening the Reporting of Observational Studies in Epidemiology (STROBE) statement checklist**
- **Supplementary Data 1: Additional details on study methods**
- **Supplementary Figure 1: Directed acyclic graph of home-learning environment and child outcomes**
- **Supplementary Table 1: Characteristics of MAASTHI cohort participants (followed up during COINCIDE versus not followed up)**
- **Supplementary Table 2: Family care indicators (FCI) among children in the MAASTHI cohort**
- **Supplementary Table 3: Association of the home-learning environment with children’s cognitive, early language, and numeracy outcomes**
